# The depth of perineural invasion is an independent prognostic factor for stage II colorectal cancer

**DOI:** 10.1186/s12885-024-12206-9

**Published:** 2024-04-08

**Authors:** Hao Chen, Chao Wang, Zexian Chen, Tianze Huang, Yanyun Lin, Junguo Chen, Bin Zhang, Xiaosheng He

**Affiliations:** 1 Department of General Surgery (Colorectal Surgery), The Sixth Affiliated Hospital, Sun Yat-Sen University, Guangzhou, Guangdong 510655 China; 2 Guangdong Provincial Key Laboratory of Colorectal and Pelvic Floor Diseases, The Sixth Affiliated Hospital, Sun Yat-Sen University, Guangzhou, Guangdong 510655 China; 3 Biomedical Innovation Center, The Sixth Affiliated Hospital, Sun Yat-Sen University, 26 Yuancun Erheng Road, Guangzhou, Guangdong 510655 China; 4 Department of Pathology, The Sixth Affiliated Hospital, Sun Yat-Sen University, Guangzhou, Guangdong 510655 China

**Keywords:** Perineural invasion, Colorectal cancer, Survival

## Abstract

**Background:**

Perineural invasion (PNI) is the invasion of nerves by cancer cells and is associated with poor survival in stage II colorectal cancer. However, PNI can be further subdivided according to the depth of invasion, and the depth of PNI has not been clearly linked to prognosis.

**Method:**

This study aimed to assess the prognostic value of different depths of PNI in stage II colorectal cancer. We defined PNI in the submucosal plexus and myenteric plexus as superficial perineural invasion (sup-PNI) and PNI in the subserous plexus as deep perineural invasion (deep-PNI). Patients were divided into three groups based on the depth of PNI: sup-PNI, deep-PNI and non-PNI. Then, univariate and multivariate Cox regression analyses were conducted to evaluate the role of PNI in the prognosis of stage II colorectal cancer.

**Results:**

This study enrolled 3508 patients with stage II colorectal cancer who underwent resection for primary colorectal lesions between January 2013 and September 2019. Clinicopathological features, including elevated carcinoembryonic antigen (CEA) levels, T4 stage, poor differentiation, deficient DNA mismatch repair (dMMR), and vascular invasion, were correlated with deep-PNI. Multivariate analyses revealed that deep-PNI was associated with worse overall survival (OS; hazard ratio [HR], 3.546; 95% confidence interval [CI], 2.307–5.449; *P* < 0.001) and disease-free survival (DFS; HR, 2.921; 95% CI, 2.032–4.198; *P* < 0.001), compared with non-PNI. Conversely, no significant difference in OS or DFS was observed between the sup-PNI and non-PNI groups in multivariate analyses.

**Conclusions:**

The study demonstrated that the depth of PNI was an independent prognostic factor for patients with stage II colorectal cancer, and patients with deep PNI had a worse prognosis. Thus, patients with PNI require further subdivision according to the depth of invasion.

**Supplementary Information:**

The online version contains supplementary material available at 10.1186/s12885-024-12206-9.

## Introduction

Colorectal cancer (CRC) is the third most common cancer and second leading cause of cancer-related deaths worldwide [1]. Perineural invasion (PNI) is a common route of cancer spread in malignant diseases [2, 3]. Large-scale studies have identified PNI as a key pathological feature that adversely affects outcomes in both colon and rectal cancer [4–7]. PNI is defined as cancer cell invasion occurring within any layer of the nerve sheath or around the perineural space, at least one-third of the nerve circumference [8]. However, the current diagnostic criteria for PNI are limited, and PNI can be further subdivided based on the location within the three types of plexuses in the gut, namely the submucosal, myenteric, and subserous plexus, based on their anatomical structure [9]. Although previous studies have explored the effect of location-specific PNI on outcomes [10–13], these studies had the following limitations: (1) including CRC patients of various stages, (2) a small sample size of PNI patients, and (3) focusing on a mixture of factors beyond PNI, which could underestimate the role of PNI during stage II CRC. In particular, the presence of PNI indicates chemotherapy for stage II CRC [14, 15]. Thus, it is important to know how the depth of PNI affects the prognosis of stage II CRC who might need receive strict surveillance or further treatment. This study aimed to examine the relationship between the depth of PNI and prognosis in stage II CRC, which could discriminate these specific patients to receive strict surveillance or further chemotherapy.

## Methods

### Patients and data collection

This retrospective study was conducted at The Sixth Affiliated Hospital of Sun Yat-sen University and included 3508 patients who had undergone surgical resection for stage II CRC between January 2013 and September 2019. Patients were included if they met the following criteria: (1) diagnosed with stage II CRC according to the 8^th^ edition of the American Joint Committee on Cancer (AJCC), (2) had undergone complete resection of the primary colorectal lesions, and (3) had pathology reports indicating the presence or absence of PNI. Patients were excluded if they had (1) insufficient follow-up information, (2) insufficient pathological information, (3) multiple primary malignant tumors.

All retrospective data were obtained from the Institutional Database Program of Colorectal Disease (IDPCD) at the Sixth Affiliated Hospital of Sun Yat-sen University. The following data were collected using the Electronic Medical Record System: age, sex, BMI, TNM stage (AJCC), degree of differentiation, presence of lymphovascular invasion, preoperative serum CEA levels, and harvested lymph nodes. After radical surgery, follow-up studies were performed every 3 months for 3 years, every 6 months for 5 years, and annually after 5 years, as recommended by the CSCO guidelines [16]. Follow-up included medical history, physical examination, routine blood tests, comprehensive biochemical examination, thoracic-abdominal-pelvic CT, and colonoscopy. The follow-up period ended in June 2022.

### Histopathology

**Fig. 1:**
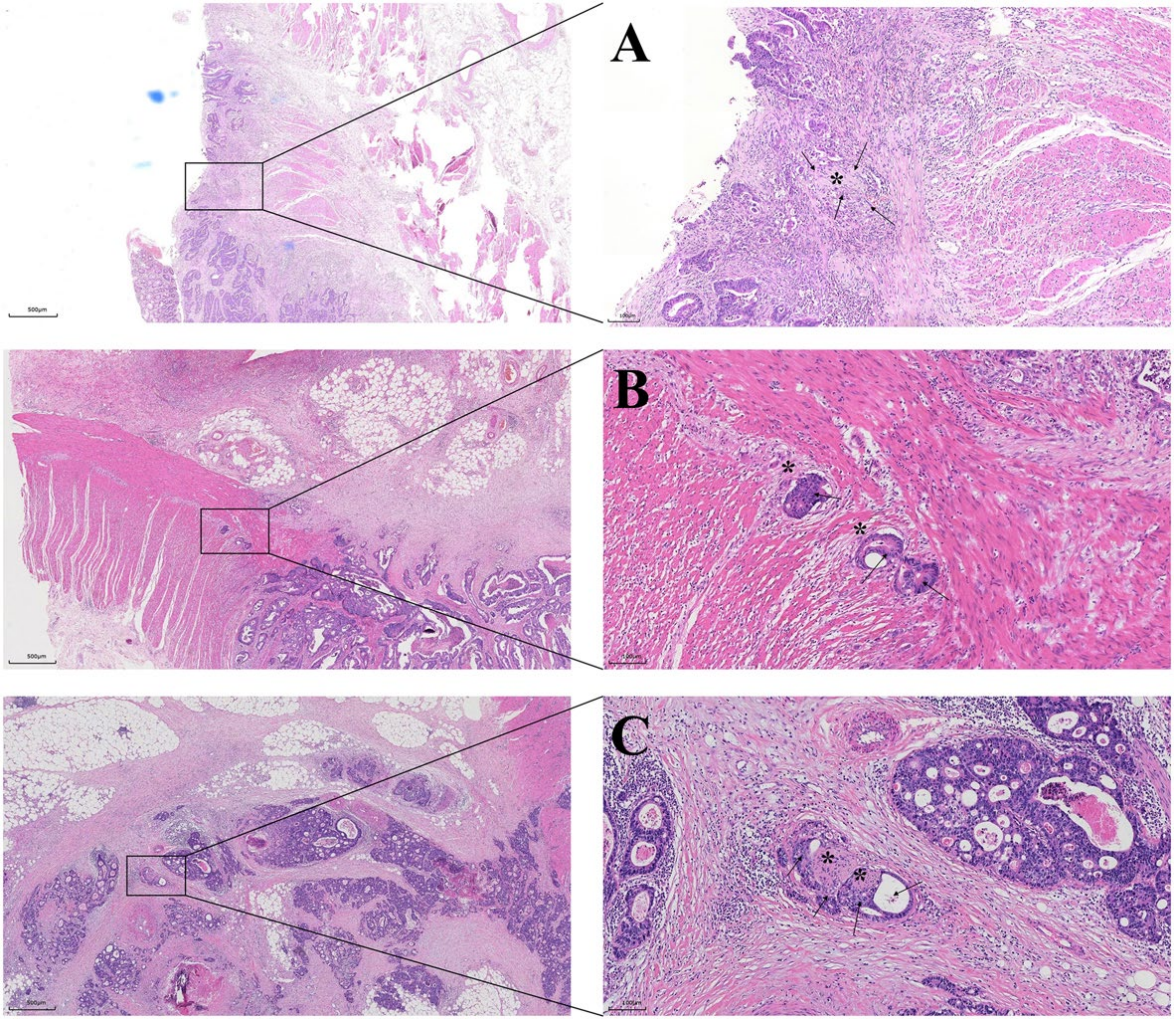
Hematoxylin and eosin staining of three types of perineural invasion. The arrow represents the tumor, and * represents the nerve. **A** PNI in the submucosal plexus. **B** PNI in the myenteric plexus. **C** PNI in the subserous plexus

For each patient included in the study, original hematoxylin and eosin (H&E)-stained slides of the tumor (average 8 slides per case; range 2–20 slides per case) were collected from the pathology department. All slides containing tumor were examined by two independent observers (H.C. and B.Z.) and reviewed by an experienced faculty pathologist (C.W.). In instances where there was any controversy in the results among the observers, the pathologist would make a collective conclusion. PNI was defined as cancer cell invasion occurring within any layer of the nerve sheath or around the perineural space, or at least one-third of the nerve circumference. Three types of PNI were differentiated based on anatomical position, namely PNI in the submucosal plexus, PNI in the myenteric plexus, and PNI in the subserous plexus, as shown in Fig. 1. Patients with PNI in the submucosal plexus and those with PNI in the myenteric plexus were grouped as the superficial PNI (sup-PNI) group, while those with PNI in the subserous plexus were classified as the deep PNI (deep-PNI) group. Cases with both sup-PNI and deep-PNI were defined as deep-PNI.

### Statistical analysis

In our study, overall survival (OS) was defined as the time from surgery to death due to any cause. Disease-free survival (DFS) was defined as the time interval between surgery and the date of imaging/endoscopic testing, revealing the presence of recurrence or death due to any cause. The baseline characteristics were compared using Fisher’s exact test and Pearson’s chi-square test, depending on the nature of the data. Survival analysis was performed using the Kaplan-Meier method and the Cox proportional hazards model. The association of baseline characteristics with OS and DFS was first assessed using univariate Cox analysis, and parameters with *P* < 0.05 were included in the final multivariate Cox regression model. Statistical significance was set at *P* < 0.05. All statistical analyses were performed using the SPSS software (version 26.0; IBM, Armonk, NY, USA).

## Results

### Patient characteristics

A total of 3508 stage II CRC patients were included in the study, as shown in Supplementary Fig. 1. Of these, 245 (7.0%) were classified into the sup-PNI group, 67 (1.9%) into the deep-PNI group, and 3196 (91.1%) into the non-PNI group. Table 1 summarizes the baseline characteristics of the patients stratified by the depth of PNI. Notably, patients with deep-PNI were more likely to exhibit elevated CEA levels, T4 stage, poor differentiation, dMMR, and vascular invasion (*P* < 0.001).

**Table 1:** Baseline characteristics of patients recruited in this study

	**Non-PNI group** (*N* = 3196)	**sup-PNI group** (*N* = 245)	**deep-PNI group** (*N* = 67)	**Total** (*N* = 3508)	***P* value**
**Gender, n(%)**					0.363
Female	1163(36.4)	98(40.0)	28(41.8)	1289(36.7)	
Male	2033(63.6)	147(60.0)	39(58.2)	2219(63.3)	
**Age, n(%)**					0.031
≥ 50	2643(82.7)	217(88.6)	52(77.6)	2912(83.0)	
< 50	553(17.3)	28(11.4)	15(22.4)	596(17.0)	
**BMI, n(%)**					0.410
≤ 24	2182(68.4)	162(66.1)	50(74.6)	2394(68.3)	
> 24	1009(31.6)	83(33.9)	17(25.4)	1109(31.7)	
**CEA, n(%)**					< 0.001
≤ 5	2084(65.5)	148(60.9)	25(37.3)	2257(64.6)	
> 5	1099(34.5)	95(39.1)	42(62.7)	1236(35.4)	
**Tumor Location, n(%)**					< 0.001
Colon	1890(59.1)	182(74.3)	40(59.7)	2112(60.2)	
Rectum	1306(40.9)	63(25.7)	27(40.3)	1396(39.8)	
**T, n(%)**					< 0.001
T3	2912(91.1)	218(89.0)	41(61.2)	3171(90.4)	
T4	284(8.9)	27(11.0)	26(38.8)	337(9.6)	
**Histology,n(%)**					0.001
Adenocarinoma	2953(92.5)	243(99.2)	62(92.5)	3258(92.9)	
Other^a^	241(7.5)	2(0.8)	5(7.5)	248(7.1)	
**Differentiation, n(%)**					0.006
Poor	366(11.5)	19(7.8)	12(17.9)	397(11.3)	
Median	2244(70.3)	197(80.4)	45(67.2)	2486(70.9)	
Well	584(18.3)	29(11.8)	10(14.9)	623(17.8)	
**MMR status, n(%)**					< 0.001
pMMR	2750(87.7)	240(99.2)	64(95.5)	3054(88.7)	
dMMR	384(12.3)	2(0.8)	3(4.5)	389(11.3)	
**Vascular Invasion, n(%)**					< 0.001
No	3041(95.3)	207(84.5)	54(80.6)	3302(94.2)	
Yes	151(4.7)	38(15.5)	13(19.4)	202(5.8)	
**Harvested Lymph Nodes, n(%)**					0.261
> 12	2781(87.3)	215(87.8)	54(80.6)	3050(87.2)	
≤ 12	406(12.7)	30(12.2)	13(19.4)	449(12.8)	

*PNI* Perineural invasion, *BMI* Body mass index, *CEA* Carcinoembryonic antigen, *MMR* Mismatch repair

^a^Other includes mucinous adenocarcinoma, signet ring cell carcinoma, neuroendocrine carcinoma

### Survival outcomes of the depth of PNI in Stage II CRC patients

**Fig. 2:**
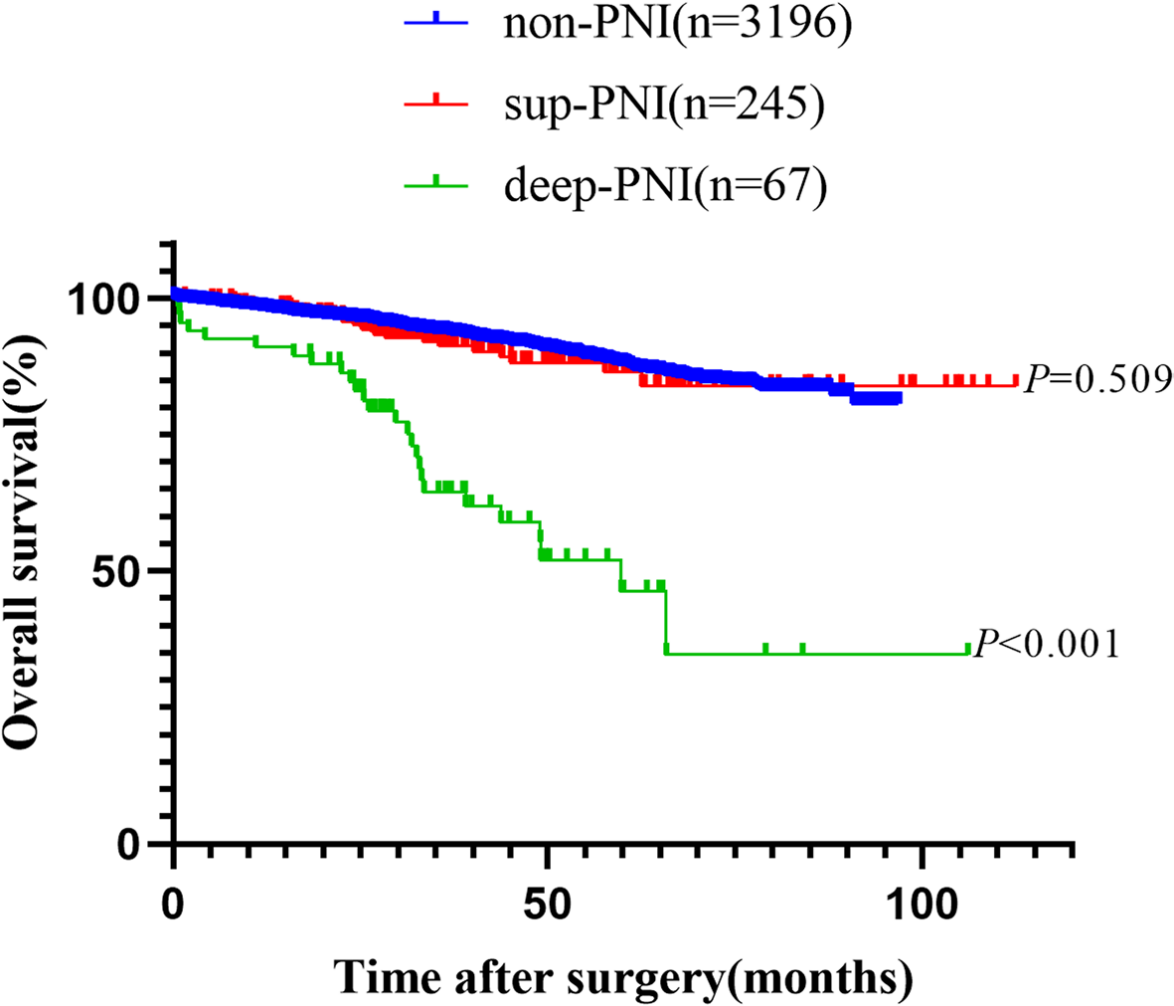
Kaplan–Meier curves of overall survival analyses

**Fig. 3:**
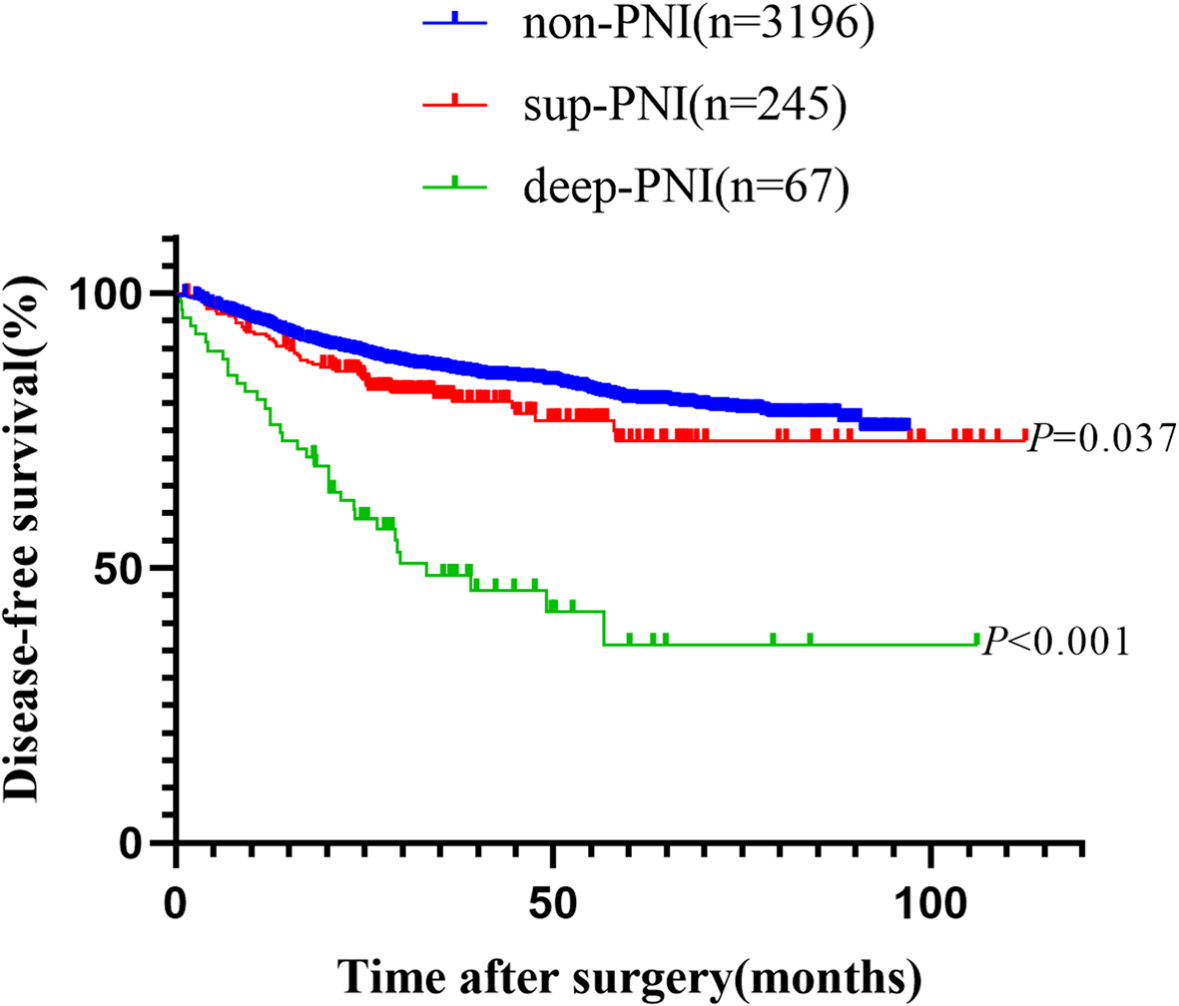
Kaplan–Meier curves of disease-free survival analyses

The median overall follow-up was 46.7 months (95% CI, 45.8–47.6). The 5-year OS rates in the non-PNI, sup-PNI, and deep-PNI groups were 87.8%, 86.4%, and 46.2%, respectively, as shown in Fig. 2. Similarly, the 5-year DFS rates in the non-PNI, sup-PNI, and deep-PNI groups were 80.3%, 73.2%, and 36.1%, respectively, as demonstrated in Fig. 3. When comparing the sup-PNI group and non-PNI group, univariate analysis showed no significant difference in OS (*P* = 0.509), but DFS demonstrated a statistical difference (*P* = 0.037). Nevertheless, the multivariate analysis did not reveal any statistical difference in OS (*P* = 0.939) or DFS (*P* = 0.140) between the two groups in Table 2. After separating colon and rectal cancer, similar results were showed in Stage II colon or rectal patients in Supplementary Fig. 2.

**Table 2:** Univariate and multivariate Cox models evaluated the effect of the depth of PNI on OS and DFS in Stage II CRC patients

	**Univariate Cox model**		**Multivariate Cox model**	
	**Hazard Ratio (95% CI)**	***P*-value**	**Hazard Ratio (95% CI)**	***P*-value**
**OS: the depth of PNI**^**a**^				
Non-PNI				
sup-PNI	1.154(0.755–1.764)	0.509	1.017(0.654–1.583)	0.939
deep-PNI	5.505(3.685–8.224)	< 0.001	3.546(2.307–5.449)	< 0.001
**DFS: the depth of PNI**^**b**^				
Non-PNI				
sup-PNI	1.366(1.019–1.831)	0.037	1.258(0.928–1.706)	0.140
deep-PNI	4.360(3.095–6.142)	< 0.001	2.921(2.032–4.198)	< 0.001

Parameters with *P* values < 0.05 in the univariate Cox model were then entered into a final multivariable Cox regression model. Detailed data are shown in Supplementary Table 1 and Table 2

^a^Adjusted for the depth of PNI, Gender, Age, BMI, CEA, T4, Differentiation, dMMR, Vascular Invasion, Harvested Lymph Nodes

^b^Adjusted for the depth of PNI, Gender, Age, CEA, Tumor Location, T4, Differentiation, dMMR, Vascular Invasion, Harvested Lymph Nodes

Univariate and multivariate analysis for OS was displayed in Table 2 and Supplementary Table 1. The univariate analysis demonstrated that patients in the deep-PNI group exhibited significantly worse OS compared to those in the non-PNI and sup-PNI groups (HR, 5.505; 95% CI, 3.685–8.224; *P* < 0.001). Furthermore, the multivariate analysis discovered that deep-PNI was an independent predictor of OS (HR, 3.546; 95% CI, 2.307–5.449; *P* < 0.001). Additional independent prognostic predictors of OS included age < 50, male, BMI ≤ 24, elevated CEA and T4 levels, poor differentiation, dMMR, vascular invasion, and the number of harvested lymph nodes ≤ 12.

The survival analysis for DFS was presented in Table 2 and Supplementary Table 2. The univariate analysis revealed that the deep-PNI group had a worse DFS (HR, 4.360; 95% CI, 3.095–6.142; *P* < 0.001). In addition, the multivariate analysis highlighted that deep-PNI was an independent predictor of DFS (HR, 2.921; 95% CI, 2.032–4.198; *P* < 0.001). Other independent prognostic predictors were rectal cancer, T4 stage, poor differentiation, dMMR, vascular invasion, and the number of harvested lymph nodes ≤ 12.

Prognostic analyses for T3 and T4 stages separately were displayed in Supplementary Fig. 3. The results indicated that the depth of PNI is a prognostic factor for OS and DFS in both T3 and T4 stage. And the multivariate analysis in Supplementary Tables 3 and 4 revealed that deep-PNI emerges as an independent prognostic indicator for both OS and DFS in T3 stage, and an independent prognostic factor for OS in T4 stage.

### Influence of the depth of invasion in PNI patients

To evaluate the impact of the depth of invasion on patients with PNI, a survival analysis of the sup-PNI and deep-PNI groups was conducted, and the results presented in Table 3. Univariate analysis showed that deep PNI was a high-risk factor for OS (HR, 4.688; 95% CI, 2.673–8.222; *P* < 0.001) and DFS (HR: 3.171; 95% CI, 1.669–6.025; *P* < 0.001), compared to sup PNI. In the multivariate analysis, which was adjusted for other prognostic factors such as CEA, tumor location, T4, differentiation, dMMR, and vascular invasion, the depth of PNI was identified as an independent prognostic factor for OS (HR, 3.181; 95% CI, 2.059–4.914; *P* < 0.001) and DFS (HR, 2.817; 95% CI, 1.744–4.549; *P* < 0.001).

**Table 3:** Univariate and multivariate Cox models evaluated the effect of the depth of PNI on OS and DFS in Stage II CRC patients with PNI

	**Univariate Cox model**		**Multivariate Cox model**	
	**Hazard Ratio (95% CI)**	***P*-value**	**Hazard Ratio (95% CI)**	***P*-value**
**OS: the depth of PNI**^**a**^				
sup-PNI				
deep-PNI	4.688(2.673–8.222)	< 0.001	3.171(1.669–6.025)	< 0.001
**DFS: the depth of PNI**^**b**^				
sup-PNI				
deep-PNI	3.181(2.059–4.914)	< 0.001	2.817(1.744–4.549)	< 0.001

^a^Adjusted for the depth of PNI, CEA, Tumor Location, T4, Differentiation, dMMR, Vascular Invasion

^b^Adjusted for the depth of PNI, T4, Differentiation, Vascular Invasion

### Influence of the depth of invasion in recurrence and metastasis

Recurrence and metastasis with the depth of PNI were represented in Supplementary Table 5. The non-PNI group, sup-PNI group, and deep-PNI group showed 77 cases (2.4%), 3 cases (1.2%), and 3 cases (4.5%) of recurrence, respectively, with no significant difference among the three groups (*P* = 0.260).

There were 294 cases (9.2%), 29 cases (11.8%), and 18 cases (26.9%) of postoperative metastasis in the non-PNI group, sup-PNI group, and deep-PNI group, respectively. With increasing depth of PNI, there was a significant increase in metastasis (*P* < 0.001). After subdividing the metastatic sites in Supplementary Table 6, it was found that the incidence of liver metastasis was relatively higher in the sup-PNI group. And the incidence of lung and other metastases was higher in the deep-PNI group.

To further investigate the risk factors for metastasis, we constructed a logistic regression model in Supplementary Table 7. Univariate analysis showed that compared to non-PNI, deep-PNI significantly increased the risk of metastasis. Multivariate analysis further confirmed that deep-PNI is an independent risk factor for metastasis in stage II CRC patients. Other independent risk factors included T4 stage, poorly differentiated tumors, pMMR status, and harvested lymph nodes ≤ 12.

## Discussion

PNI has been identified as one of the factors contributing to poor prognosis in stage II colorectal cancer [15–18]. However, few studies have focused on the differences in PNI based on the location of invasion within the intestinal tract. The intestinal tract comprises various layers, including the mucosal, muscular, and serosal layers, each containing different nerve plexuses with distinct shapes and functions [9]. Thus, when PNI occurs in different nerve plexuses, there may be variations in prognosis [12]. Therefore, we selected patients with stage II CRC to investigate the correlation between the depth of PNI and prognosis as well as to explore the differences in PNI based on its location within the different layers of the intestinal tract.

Upon subdividing patients with PNI into sup-PNI and deep-PNI groups, we observed that those in the deep-PNI group had a poorer prognosis compared to the sup-PNI group. This finding implies that infiltration of cancer cells into the subserous plexus may result in worse outcomes. In contrast, there was no statistical difference in survival between patients in the sup-PNI group and those in the non-PNI group, indicating that sup-PNI may not be a predictive factor for survival. Given that current guidelines recommend adjuvant therapy for patients with PNI [15, 16], patients with sup-PNI may be subjected to overtreatment as their survival rates are similar to those without PNI. Therefore, it is important to supplement the current PNI definition with the depth of invasion to provide a more accurate prognosis and treatment recommendation. And there is an urgent need for a new nerve-targeted therapy to improve the prognosis of stage II CRC patients with PNI, such as targeting CD51, CD74 and nerve growth factor [19–22].

Current guidelines and studies recommend PNI leads to a worse prognosis in stage II CRC [23–25], but do not distinguish the depth of PNI. We conducted a survival analysis that included several well-established high-risk factors for stage II CRC, such as T4, poor differentiation, vascular invasion, and the number of harvested lymph nodes < 12 [4, 15]. In the multivariate analysis of OS and DFS, we found that the depth of PNI was an independent prognostic factor. Thus, it may be necessary to revise the definition of high-risk factors for stage II CRC in the existing guidelines by specifying deep PNI, which could help distinguish the prognosis of patients.

The present study had some limitations that should be taken into account when interpreting the results. Firstly, as a single-center retrospective study, our findings may be limited by potential information bias and may not be generalizable to other populations. Secondly, we did not conduct immunostaining for neuropeptide markers in patient specimens [26], which may have affected the accuracy of the PNI classifications. Finally, due to the insufficient number of dMMR patients, it is difficult to explain the relationship between dMMR and PNI, and the relevant study is being conducted. Despite these limitations, our study highlights the importance of differentiating PNI based on the depth of invasion and suggests that the current definition of PNI needs to be revised as PNI in the subserous plexus.

## Conclusions

In conclusion, our study demonstrates that the depth of PNI is a significant independent prognostic factor for recurrence and survival in patients with stage II CRC. Specifically, patients with PNI in the subserous plexus had a worse prognosis, while the difference in prognosis between stage II CRC patients with PNI in the submucosal and myenteric plexuses and those without PNI was not significant. These findings suggest that the definition of PNI in high-risk factors should be revised to focus on PNI in the subserous plexus.

### Supplementary Information

**Additional file 1: Supplementary Figure 1.** Flow Chart of this study.

**Additional file 2: Supplementary Figure 2.** Kaplan–Meier curves of survival analyses. Overall survival and disease-free survival in colon and rectum cancer according to the depth of PNI. OS and DFS were significantly different in three groups. A and B were survival curves of colon cancer, and C and D were rectum cancer.

**Additional file 3: Supplementary Figure 3.** Kaplan–Meier curves of survival analyses in T3 and T4 stages separately. Overall survival and disease-free survival in T3 and T4 stage according to the depth of PNI. OS and DFS were significantly different in three groups. A and B were survival curves of T3 stage, and C and D were T4 stage.

**Additional file 4: Supplementary Table 1.** Univariate and multivariate Cox models for overall survival of each characteristic.

**Additional file 5: Supplementary Table 2.** Univariate and multivariate Cox models for disease-free survival of each characteristic.

**Additional file 6: Supplementary Table 3.** Univariate and multivariate Cox models evaluated the effect of the depth of PNI on OS and DFS in T3 stage patients.

**Additional file 7: Supplementary Table 4.** Univariate and multivariate Cox models evaluated the effect of the depth of PNI on OS and DFS in T4 stage patients.

**Additional file 8: Supplementary Table 5.** The association between the depth of PNI and recurrence, metastasis in stage II CRC patients.

**Additional file 9: Supplementary Table 6.** The association between the depth of PNI and the site of metastasis in stage II CRC patients.

**Additional file 10: Supplementary Table 7.** Univariate and multivariate logistics models evaluated the effect of the depth of PNI on metastasis in stage II CRC patients.

## Data Availability

The data supporting the findings of this study are available from the corresponding author upon reasonable request.
