# Supplementary figures and images for: The depth of perineural invasion is an independent prognostic factor for stage II colorectal cancer
Source: BMC Cancer. 2024 Apr 8;24:433. doi: 10.1186/s12885-024-12206-9 (PMC11003015; doi:10.1186/s12885-024-12206-9)

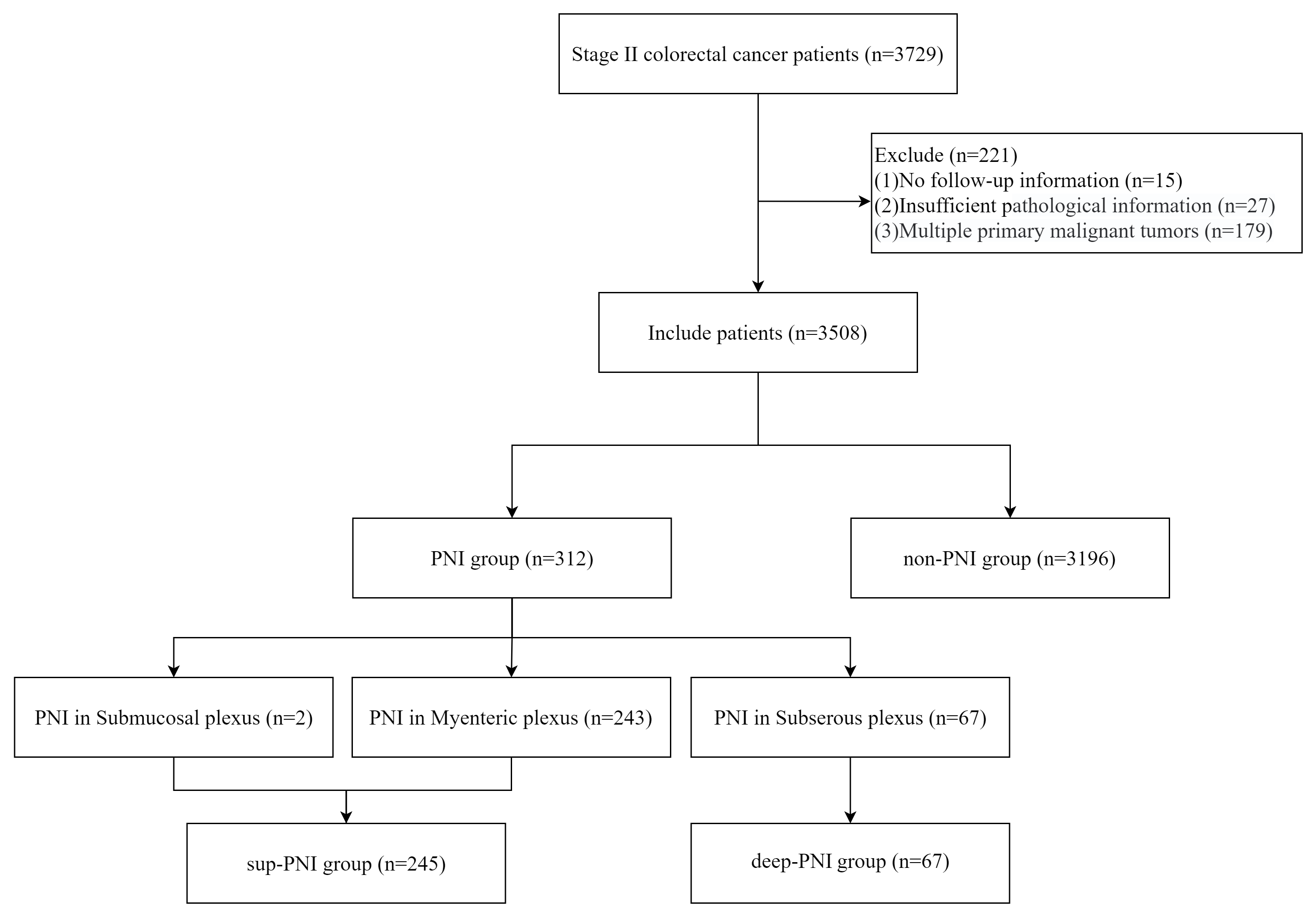

Supplement: Supplementary file 1 — Additional file 1: Supplementary Figure 1. Flow Chart of this study. [file 12885_2024_12206_MOESM1_ESM.png]

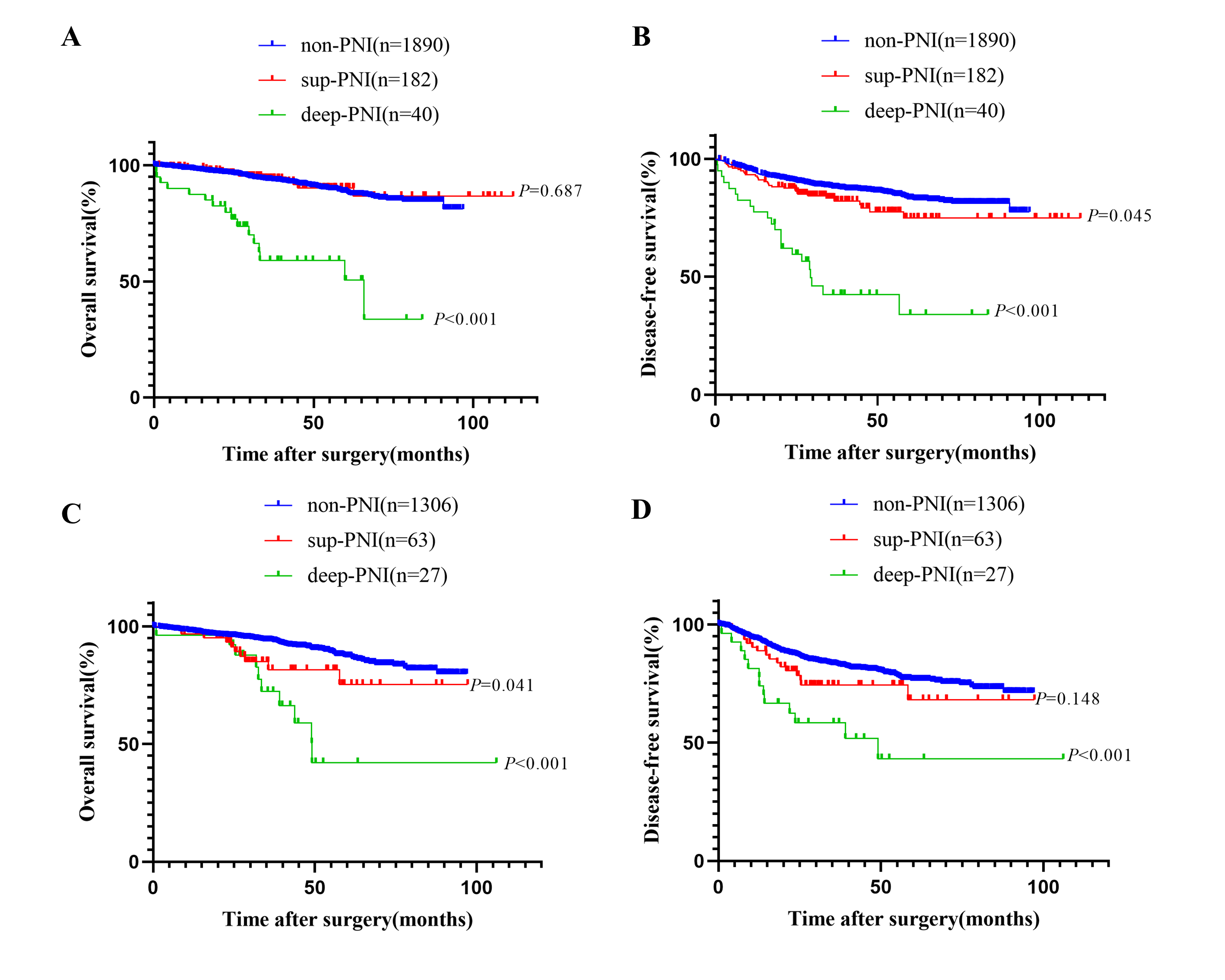

Supplement: Supplementary file 2 — Additional file 2: Supplementary Figure 2. Kaplan–Meier curves of survival analyses. Overall survival and disease-free survival in colon and rectum cancer according to the depth of PNI. OS and DFS were significantly different in three groups. A and B were survival curves of colon cancer, and C and D were rectum cancer. [file 12885_2024_12206_MOESM2_ESM.png]

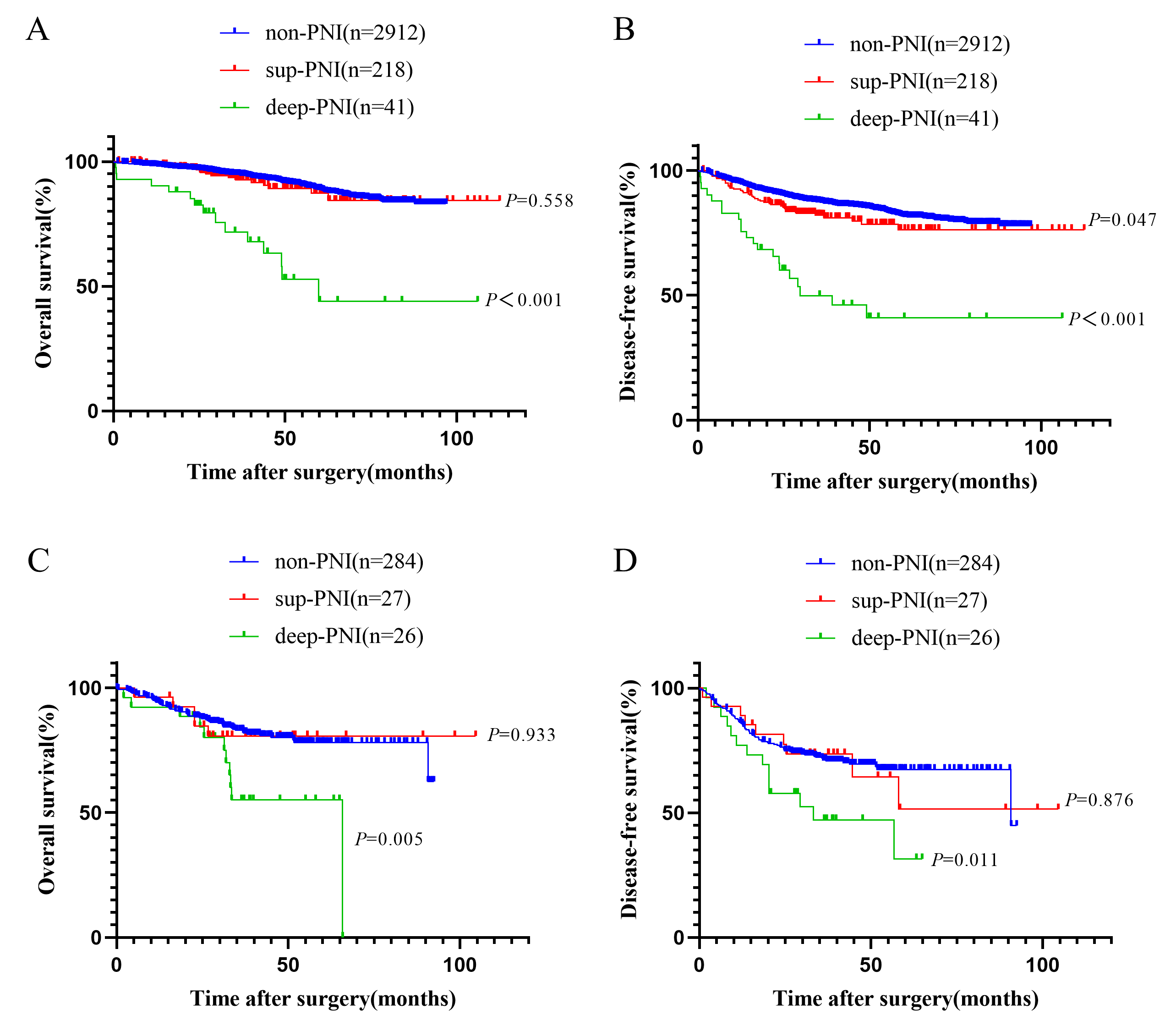

Supplement: Supplementary file 3 — Additional file 3: Supplementary Figure 3. Kaplan–Meier curves of survival analyses in T3 and T4 stages separately. Overall survival and disease-free survival in T3 and T4 stage according to the depth of PNI. OS and DFS were significantly different in three groups. A and B were survival curves of T3 stage, and C and D were T4 stage. [file 12885_2024_12206_MOESM3_ESM.png]
